# Predicting 1-year mortality of patients with diabetes mellitus in Kazakhstan based on administrative health data using machine learning

**DOI:** 10.1038/s41598-023-35551-4

**Published:** 2023-05-24

**Authors:** Aidar Alimbayev, Gulnur Zhakhina, Arnur Gusmanov, Yesbolat Sakko, Sauran Yerdessov, Iliyar Arupzhanov, Ardak Kashkynbayev, Amin Zollanvari, Abduzhappar Gaipov

**Affiliations:** 1 Department of Electrical and Computer Engineering, School of Engineering and Digital Sciences, Nazarbayev University, Kabanbay Batyr Avenue 53, Astana, Kazakhstan; 2 Department of Medicine, School of Medicine, Nazarbayev University, Kerey and Zhanibek Khans Street 5/1, Astana, Kazakhstan; 3 Department of Mathematics, Nazarbayev University, Kabanbay Batyr Avenue 53, Astana, Kazakhstan

**Keywords:** Machine learning, Predictive medicine, Risk factors, Epidemiology

## Abstract

Diabetes mellitus (DM) affects the quality of life and leads to disability, high morbidity, and premature mortality. DM is a risk factor for cardiovascular, neurological, and renal diseases, and places a major burden on healthcare systems globally. Predicting the one-year mortality of patients with DM can considerably help clinicians tailor treatments to patients at risk. In this study, we aimed to show the feasibility of predicting the one-year mortality of DM patients based on administrative health data. We use clinical data for 472,950 patients that were admitted to hospitals across Kazakhstan between mid-2014 to December 2019 and were diagnosed with DM. The data was divided into four yearly-specific cohorts (2016-, 2017-, 2018-, and 2019-cohorts) to predict mortality within a specific year based on clinical and demographic information collected up to the end of the preceding year. We then develop a comprehensive machine learning platform to construct a predictive model of one-year mortality for each year-specific cohort. In particular, the study implements and compares the performance of nine classification rules for predicting the one-year mortality of DM patients. The results show that gradient-boosting ensemble learning methods perform better than other algorithms across all year-specific cohorts while achieving an area under the curve (AUC) between 0.78 and 0.80 on independent test sets. The feature importance analysis conducted by calculating SHAP (SHapley Additive exPlanations) values shows that age, duration of diabetes, hypertension, and sex are the top four most important features for predicting one-year mortality. In conclusion, the results show that it is possible to use machine learning to build accurate predictive models of one-year mortality for DM patients based on administrative health data. In the future, integrating this information with laboratory data or patients’ medical history could potentially boost the performance of the predictive models.

## Introduction

The burden of diabetes is a rising concern in healthcare worldwide. According to the estimates of the International Diabetes Federation (IDF), in 2021, there were 537 million adults living with diabetes, which is 6.79% of the world’s population^1^. According to the global epidemiological data from 2017, the predicted number of diabetes will be 693 million by 2030, while the most recent study projects a rise up to 783 million by 2045. IDF study reports that 75% of cases live in low- and middle-income countries, and there were 6.7 million deaths worldwide in 2021^2^. In Kazakhstan, overall 472,950 people were listed with Type 1 and Type 2 Diabetes in inpatient and outpatient registries in the same period^3^.

Diabetes mellitus (DM) affects the quality of life and leads to disability, high morbidity, and premature mortality. DM is a risk factor for cardiovascular, neurological, and renal diseases, and places a major burden on healthcare systems globally. At the same time, populations around the world are rapidly aging, and that further contributes to a higher number of incident DM cases. Elderly people can have multiple comorbidities and complications along with DM, which elevates mortality rates^4^. In this regard, predicting the one-year mortality of patients with DM is at the core of health management systems as it can help clinicians tailor treatments to improve the survival of DM patients.

In recent years, constructing data-driven predictive models using machine learning has found various applications in health care^5,6^. In^7^, Random Forest (RF) algorithm was used for the early prediction of diabetes using a number of variables such as regular and ultralente insulin dose, socio-demographic factors, and hypoglycemic symptoms, to just name a few. In another study^8^, different predictive models were examined to predict diabetes based on several factors such as glucose level, blood pressure, and insulin. Moreover, machine learning techniques were used to predict the mortality of diabetes patients based on HbA1c and lipid parameters^9^. With successful applications of machine learning in disease and mortality prediction, it is highly anticipated that it can be used to predict the one-year mortality of patients with DM based on ordinary clinical variables. Some mortality prognostic models have been developed using machine learning approaches on clinical and administrative data^9–11^. Furthermore, several studies have attempted to predict mortality for DM patients in an intensive care unit (ICU)^12–15^. However, predicting the one-year mortality of DM patients based solely on administrative health data including diagnoses, comorbidities, procedures, and demographics have not been used before. This is in sharp contrast with the previous studies where additional information including the results of laboratory tests or vital signs (e.g., ICU admission) were used for prediction.

In this regard, we used the Unified National Electronic Health System (UNEHS) of Kazakhstan to collect ordinary clinical data for a large cohort of DM patients who registered in hospitals across the country between January 2014 and December 2019. The detailed description of database is given elsewhere^16^. The collected data was then divided into four subcohorts to predict mortality within a year (starting from 2016) based on collected clinical data up to the end of the preceding year. We then develop a comprehensive machine learning platform to construct one predictive model of one-year mortality for each subcohort. Our study points to the feasibility and robustness of the developed machine learning (ML) platform for predicting the one-year mortality of DM patients in Kazakhstan using aggregated nationwide administrative healthcare data. We also identify and rank the importance of clinical variables that were used by the constructed predictive models of mortality.

To our knowledge, there is a lack of models that can distinguish high-risk populations and forestall the mortality of individuals with diabetes in Central Asian countries. The development of a prognostic model for one-year mortality in diabetes mellitus has the potential to assist healthcare practitioners in devising individualized treatment plans and interventions that can mitigate adverse consequences. Furthermore, this could aid in the allocation of resources, as patients who are deemed high-risk may necessitate more frequent monitoring or follow-up care.

## Results

### Data description

The objective of this study is to predict one-year mortality in DM patients based on administrative health data. In this regard we collected clinical data for patients diagnosed with DM from UNEHS^3^, which is a nationwide electronic health record repository of patients admitted to hospitals across Kazakhstan between mid-2014 and December 2019. After excluding patients with the missing outcome, which is the mortality with possible values being dead or alive, the data was divided into four yearly-specific cohorts to predict mortality within a specific year based on clinical information collected up to the end of the preceding year. Hereafter, these subcohorts are referred to as 2016-, 2017-, 2018-, and 2019-cohorts and contain 262,212, 301,563, 337,846, and 370,807 patients, respectively. For example, the cohort of 2018 contains only patients who have been admitted to the hospital and were alive on or prior to 31st December 2017 and, at the same time, the value of the outcome variable in 2018 is known (see Supplementary materials for more details). The data is highly imbalanced with the ratio of death to alive being, 10,490:251,722, 11,568:289,995, 13,168:324,678, 13,534:357,273, for 2016-, 2017-, 2018-, and 2019-cohorts, respectively. The clinical variables used as predictors of mortality in the collected cohorts are listed in Table 1 (more information in the Supplementary Table S1). The missing values of numeric and categorical predictors were imputed based on the median and mode of those variables in training data, respectively. We used a stratified random split to divide each yearly-specific cohort with an 80/20 ratio into training and test sets. The stratification is performed to keep the proportion of samples that appear in training and test sets the same as the full cohort. The training set is used for predictive model (classifier) training and selection, while the test set is used for model evaluation. Each year-specific constructed predictive model classifies the status of the patient (dead or alive) within the next year after collecting the patients' clinical data.

**Table 1:** Name and description of predictors (features) used in yearly-specific cohorts.

Feature	Description	Unit	Type
Age	Age at the first hospitalisation recorded in the database	Years	Numeric
Sex	Gender: female or male	Binary	Categorical
Ethnicity	Three major categories: Kazakhs, Russians and others	Tertiary	Categorical
Type of Diabetes	Type of diabetes: T1D or T2D, and other types	Tertiary	Categorical
CHD	Comorbidity for diabetes (yes/no)	Binary	Categorical
CVA	Comorbidity for diabetes (yes/no)	Binary	Categorical
Neoplasms	Comorbidity for diabetes (yes/no)	Binary	Categorical
Hypertension	Comorbidity for diabetes (yes/no)	Binary	Categorical
Hospitalisation	Number of hospitalizations during observation period	Frequency	Numeric
Duration of Diabetes	Follow-up time until December 31 the preceding year of prediction	Years	Numeric

CHD, coronary heart disease; CVA, cerebrovascular accident; T1D, type 1 diabetes; T2D, type 2 diabetes.

### Training and selecting yearly-specific classifier of one-year mortality—model training and selection

We deployed nine classifiers, namely, Gaussian Naïve Bayes (GNB)^17^, K-nearest neighbors (KNN)^17^, logistic regression with *L*_*2*_ ridge penalty (LRR)^18^, random forest (RF)^18^, AdaBoost with decision trees (ADB)^19^, gradient boosting with regression trees (GBRT)^20^, XGBoost (XGB)^21^, linear discriminant analysis (LDA)^22^, and perceptron (PER)^17^ (see the Materials and Methods section for more details on the rationale behind the selecting these classifiers). The candidate hyperparameter space for each classifier is discussed in the Materials and Methods. The developed ML platform performs model selection (including hyperparameter tuning) using each yearly-specific training set by calculating the area under the curve (AUC) performance metric using stratified 5-fold cross-validation (5-CV). Table 2 shows the 5-fold CV estimate of the AUC for each classifier. As observed in Table 2, GBRT achieved the highest AUC for the years 2016 and 2017, while XGB showed the highest AUC for 2018 and 2019. That being said, both classifiers are from the class of gradient boosting ensemble learning. This shows the superiority of gradient-boosting ensemble learning compared with other algorithms in our application.

**Table 2:** The classifier-specific estimated AUC mean ± standard deviation over 5 folds of 5-fold cross-validation obtained on the training set.

Classifier	AUC			
	2016	2017	2018	2019
GNB	0.705 ± 0.009	0.697 ± 0.009	0.695 ± 0.009	0.703 ± 0.003
KNN	0.602 ± 0.003	0.585 ± 0.003	0.588 ± 0.003	0.584 ± 0.004
LRR	0.756 ± 0.006	0.744 ± 0.003	0.743 ± 0.002	0.749 ± 0.005
RF	0.687 ± 0.004	0.681 ± 0.009	0.682 ± 0.003	0.689 ± 0.003
ADB	0.770 ± 0.003	0.758 ± 0.005	0.755 ± 0.005	0.764 ± 0.005
XGB	0.793 ± 0.007	0.784 ± 0.002	**0.791 ± 0.003**	**0.797 ± 0.006**
GBRT	**0.795** ± **0.005**	**0.786 ± 0.007**	0.787 ± 0.002	0.794 ± 0.002
LDA	0.755 ± 0.007	0.742 ± 0.005	0.741 ± 0.005	0.748 ± 0.002
PER	0.593 ± 0.013	0.605 ± 0.012	0.641 ± 0.011	0.596 ± 0.010

The year-specific highest AUC mean and standard deviation is identified in bold.

GNB, Gaussian Naive Bayes; KNN, K-nearest neighbors; LRR, logistic regression; RF, random forest; ADB, Adaboost with decision trees; GBRT, gradient boosting with regression trees; XGB, XGBoost; LDA, linear discriminant analysis; PER, perceptron.

### Evaluating year-specific classifier of one-year mortality—model evaluation

**Figure 1:**
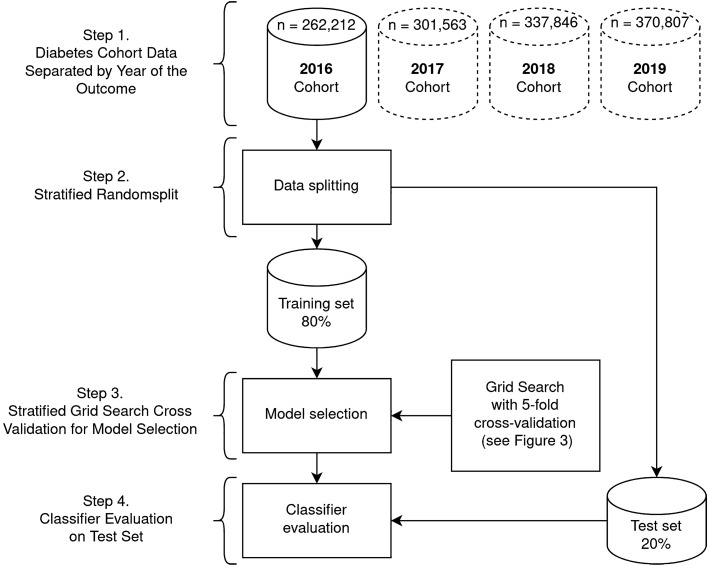
A schematic diagram of the developed machine learning platform.

The best year-specific classification algorithm and the values of its hyperparameters that were identified in the model selection phase were used to train one final year-specific classifier on the entire training set. Then each of these trained classifiers is evaluated on the corresponding (year-specific) test set using several performance metrics including AUC, balanced accuracy, sensitivity, specificity, and the geometric mean of sensitivity and specificity (G-mean). Figure 1 shows the entire process of model selection and evaluation. The results of the model evaluation are shown in Table 3. The confusion matrices across all year-specific test sets are presented in Supplementary Materials (Supplementary Tables S2–S5). All classifiers achieved an AUC greater than 0.78, which is ranked ‘fair’ (close to ‘good’) as per objective metrics of diagnostic tests (see^23^ for performance guidance based on AUC). At the same time, the estimated AUCs on test sets are quite close to the AUCs previously achieved using a 5-fold CV. This observation per se shows the robustness of developed classifiers. The results also show that the developed classifiers have a higher sensitivity than specificity. In the trade-off between sensitivity and specificity of our developed classifiers, this is indeed a desirable feature for our application, because the cost of not detecting (and no intervention thereof) a patient who will die within a year is (much) higher than a patient who is labeled as “death” but will truly survive.

**Table 3:** Performance metrics of year-specific selected classifier evaluated on the test data for the same year.

Classifier	AUC	Balanced accuracy	Sensitivity	Specificity	G-mean
2016-classifier (GBRT)	0.791	0.698	0.876	0.520	0.722
2017-classifier (GBRT)	0.787	0.690	0.879	0.502	0.779
2018-classifier (XGB)	0.787	0.674	0.899	0.499	0.775
2019-classifier (XGB)	0.799	0.644	0.937	0.352	0.777

GBRT, gradient boosting with regression trees; XGB, XGBoost.

### Impact direction and importance of each feature for predicting one-year mortality

**Figure 2:**
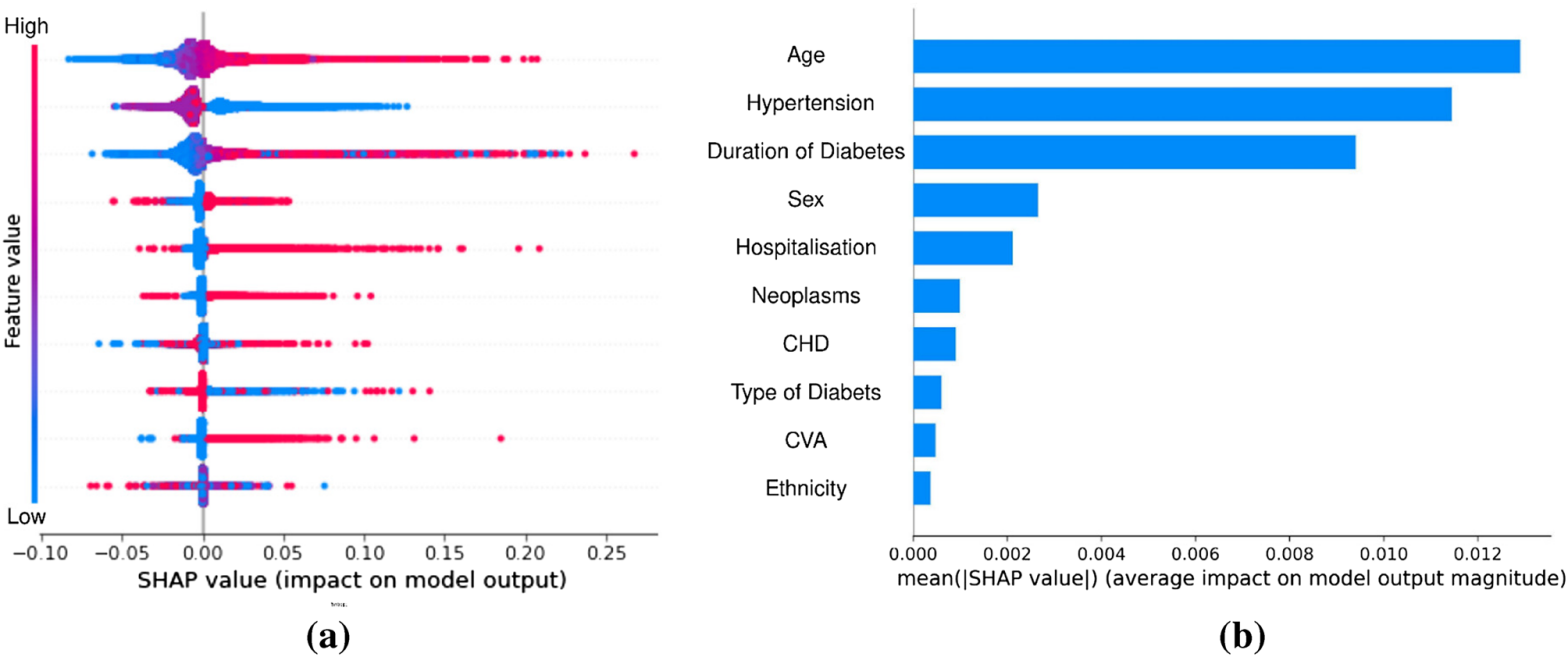
SHAP analysis of 2016-specific cohort: (**a**) SHAP summary dot plot for the 2016-specific cohort. A red dot shows a high value of the feature for a patient, whereas a blue dot shows a low value. The likelihood of mortality increases (decreases) for a positive (negative) SHAP value. Positive SHAP values for red dots show a direct dependence on the feature and the outcome, whereas the same values for blue dots imply an inverse dependence. (**b**) The mean absolute SHAP value bar plots for the 2016-specific cohort. The plot shows the feature importance on outcome prediction made by the model (a longer bar shows a more important feature).

We performed a SHAP^24^ (short for SHapley Additive exPlanations) analysis to: (1) infer the direction of impact of each feature on mortality prediction made by the year-specific model; and (2) measure the overall importance of each feature on outcome prediction. In this regard, we estimated SHAP values for the year-specific classifier that was selected in the model selection stage; that is to say, for 2016 and 2017, they were estimated for the GBRT classifier, and for 2018 and 2019, they were estimated for the XGB classifier. Furthermore, SHAP values were computed for all variables in the training dataset as no feature selection has been performed (see Discussion). Figure 2 shows the SHAP summary dot plot and mean absolute SHAP bar plot for the 2016-specific cohort. Similar plots for other year-specific classifiers are presented in Supplementary Materials. From Fig. 2a, we also observe that age and duration of diabetes are directly proportional to higher mortality. Considering binary values of hypertension, the results show that the lack of hypertension (encoded as 0) is associated with higher mortality. To summarize the results of SHAP values across all four cohorts, we determined the *average* of the mean absolute SHAP value (AMAS) for each feature across all years. This result ranks the features (from highest to lowest importance) as age, duration of diabetes, hypertension, sex, neoplasms, hospitalisation, CHD, CVA, type of diabetes, and ethnicity with AMAS being 0.0129, 0.0097, 0.0041, 0.0034, 0.0003, 0.0018, 0.0013, 0.0010, 0.0008, 0.0006, and 0.0003, respectively. Based on these findings age, duration of diabetes, hypertension, and sex are the top four most important features for predicting one-year mortality.

## Discussion

The results in Table 3 show that all trained yearly-specific classifiers achieved a predictive performance in the range of 0.78–0.799 in terms of AUC. At the same time, as per objective metrics of diagnostic tests, an estimated AUC in the range of 0.7–0.8 is generally considered a ‘fair’ predictive capacity for the test^23^.

Several studies have predicted the mortality of DM patients using a combination of clinical and administrative data. For instance, a recent study^12^ predicted the mortality of diabetic patients admitted to the ICU using nine classifiers including LR, RF, AB, XGB, GBM, artificial neural network (ANN) and majority voting. XGB and majority voting showed the best performance with an AUC of 0.867 and 0.867, respectively. Similarly, another study^13^ predicted the mortality of critically ill patients with DM using the Charlson comorbidity index (CCI), Elixhauser comorbidity index, the diabetes complications severity index (DSCI), RF, and LR as the main prediction models. The LR achieved an AUC of 0.785, while RF achieved an AUC of 0.787.

In another study^14^, the mortality of heart failure patients with diabetes was predicted using nine classifiers, including LR, RF, SVM, KNN, DT, GBM, XGBoost, LightGBM, and Bagging. The RF algorithm outperformed other algorithms, achieving an AUC of 0.92. Mortality prediction of patients with diabetes and sepsis in ICU using five classifiers were investigated in another study^15^. Authors used LR with lasso regularization, Bayes LR, decision tree, RF, and XGBoost. Out of five classifiers, the RF model showed the best performance, achieving an accuracy of 0.883. In another investigation^9^, Random Survival Forest (RSF) was used to predict the mortality of patients with diabetes and study the hazardous effects of HbA1C and lipid variability. The RSF model achieved an AUC of 0.866. Table 4 provides a summary of studies on predicting mortality of DM patients.

**Table 4:** Comparison of studies and their main predictors (important features), and model performance in terms of AUC.

Study	Dataset	Patients amount			Algorithm	Performance by AUC	Important predictors
		Alive	Dead	Imbalance proportion			
Lee, S. et al.^9^	Dataset from Hong Kong hospitals	25,186	12,372	0.491	Regularized and Weighted RSF	0.8663	Age, chronic kidney disease, baseline hemoglobin, heart failure
Barsasella, D. et al.^10^	Dataset from Taiwan NHIRD	28,510	883	0.03	RF	0.97	Displacement of lumbar intervertebral disc, cerebral artery occlusion, age, hearing loss
Ye, J. et al.^12^	MIMIC-III	8790	1164	0.132	XGBoost Majority Voting	0.86	DCSI sum, Elixhauser sum, CCI sum, mean glucose
Anand, R. S. et al.^13^	MIMIC-III	3729	382	0.102	RF and LR	0.787	Mean glucose, mean HbA1c, type of admission, severity scores
Yang, B. et al.^14^	MIMIC-IV	2815	395	0.140	RF	0.92	APS III, SOFA, urine output minimum, lactate_max
Qi, J. et al.^15^	MIMIC-IV	5000	896	0.142	RF	0.883	Lactate, age, oxygen saturation, systolic blood pressure
	eICU-CRD	715	727	0.145			
	dtChina	390	69	0.177			

MIMIC, Medical Information Mart for Intensive Care; SOFA, Sequential Organ Failure Assessment; APS III, Acute Physiology Score (APS) III; eICU-CRD, eICU Collaborative Research Database; dtChina, a large critical care database in China; NHIRD, National Health Insurance Research Database; RF, random forest.

Although our identified models have a ‘fair’ predictive capacity (close to ‘good’), their estimated AUC is generally lower than the previous studies^9,12,14,15^. This state of affairs can be attributed in part to the availability of clinical information regarding the laboratory tests and vital signs that were used in the previous investigations, whereas in our study none of these information were used.

The results of our study show that the top four most important features for predicting one-year mortality are age, duration of diabetes, hypertension, and sex. It is worthwhile to mention that throughout the work, the “importance” value of a feature is a measure of “association” between the feature and the mortality (rather than a notion of “causality”). Nonetheless, from Fig. 2a (and other similar figures in Supplementary Materials), it is observed that the age and the duration of diabetes are directly proportional to a higher mortality. Furthermore, the results show that the lack of hypertension is associated with higher mortality. In this case, hypertension has a paradoxical protective effect^3^. It can be partly explained by the reverse epidemiological phenomenon of standard risk factors in chronic diseases and chronic infections such as HIV/AIDS^25,26^. Previous studies^9–15^ have reported various predictors of mortality in diabetes; however, the identified factors have not been consistently replicated across studies, as summarized in Table 4. Age is the only predictor that was consistently shown to be significant in several studies, as well as in ours.

The association between age and diabetes mortality has been extensively studied in the literature. A number of studies have reported that increasing age is associated with a higher risk of diabetes-related mortality^27–29^. Research based on nationwide registers in Denmark showed that individuals who are diagnosed with diabetes at an older age have a higher mortality risk within the first two years after diagnosis^30^. On the other hand, another study showed that individuals diagnosed with type 2 diabetes at a younger age had a greater likelihood of mortality compared to those diagnosed at an older age^31^. Our findings indicate that elderly age at diabetes diagnosis is associated with an elevated risk of mortality.

The association between gender and diabetes mortality has been a topic of interest in recent studies, particularly in the context of gender influence on diabetes management and outcomes. The systematic review and meta-analysis conducted by Wang and colleagues showed that women with diabetes have generally a higher risk of coronary heart disease and all-cause mortality compared to men with the same condition. Specifically, women with diabetes have a 58% greater risk of CHD and a 13% greater risk of all-cause mortality^32^. Another systematic review stated that the additional likelihood of developing cancer and the higher risk of death that comes with having diabetes are slightly more pronounced in women than in men^33^. Although the majority of studies show that women with diabetes have higher risk of mortality than men with the same condition, our results indicates the opposite, which is supported by several studies^34,35^. A study from Germany found that men had a higher mortality rate associated with total T2D compared to women due to a greater relative mortality associated with undiagnosed T2D in men compared to women^35^. One possible explanation for this gender difference could be that women in Germany receive a diagnosis for T2D earlier in the course of the disease than men, which could lead to better management and outcomes. This explanation may also apply to our study, as women in Kazakhstan have greater awareness of the diabetes condition. Moreover, among older people in Kazakhstan, women had significantly higher rates of DM control (31.8%) compared to men (22.6%)^36^.

The studies from Scotland and Sweden found that among diabetic patients, women with congestive heart failure (CHF) as a comorbidity have higher mortality rate compared to men with a similar condition^37,38^. It can be related to differences in diabetes management and access to care, as well as biological factors such as hormonal changes. Moreover, type 2 diabetes is associated with a two to four-fold increase in the risk of developing CHF and ischemic stroke^39^. Numerous studies show that patients with diabetes and CHF had a significantly higher risk of all-cause mortality compared to those without CHF, even after adjusting for various clinical and demographic factors^40,41^. The increased mortality risk in patients with both CHF and diabetes may be related to impaired cardiac function, insulin resistance, and chronic inflammation. The results of the current study are consistent with the literature.

Although research shows that comorbid hypertension increases the mortality among diabetes population^42–44^, the results of this study indicate the opposite. The management of hypertension in individuals with diabetes can reduce the mortality risk by reducing the risk of developing complications related to both conditions. Effective management of hypertension can help prevent or slow the progression of damage to the blood vessels, reducing the risk of heart attack, stroke, and other cardiovascular complications^45–47^. Studies have shown that good blood pressure control can reduce the risk of cardiovascular disease and mortality in individuals with diabetes. In fact, a blood pressure goal of less than 130/80 mmHg is recommended for individuals with diabetes in order to reduce their risk of cardiovascular complications^48,49^. More profound research on this issue is needed.

The longer duration of hospitalization was significantly associated with severe complications and mortality in the Korean diabetic cohort^50^. A similar tendency was shown in the results of the current study.

Considering the relatively limited number of features (10 attributes presented in Table 1) and their administrative types, the reported range of AUC for the constructed classifiers is indeed a considerable achievement for predicting the one-year mortality of DM patients. That being said, there are a few limitations in our analysis.

From a clinical perspective, one limitation is that our data neither includes laboratory data nor patients’ medical history. In addition, the database lacks information on important comorbidities and anthropometric indices such as Alzheimer's disease, renal diseases, amputations, and BMI. Collecting and using this information would potentially boost the performance of our predictive models. Nonetheless, including this information would require running and retraining all our predictive models. At the same time, collecting further detailed patients' medical history from clinical notes available through UNEHS calls for advanced natural language processing. From a machine learning perspective, one limitation of our developed machine learning pipeline is the lack of a feature selection stage. Although this is not a critical stage in the current study due to the large sample size and a small number of features, adding laboratory data and/or the patient's medical history would possibly add a number of additional features. In that case, having a feature selection would be generally expected and help due to the curse of dimensionality in pattern recognition^17^ (also known as the peaking phenomenon^51^). We leave these investigations for future studies.

Despite the limitations of the study, there are some advantages that are noteworthy. To begin with, the data utilized in this study was derived from a population-based registry, which provides a substantial amount of information that is representative of a population of roughly half a million data points. Additionally, the data collection period was sufficiently long to encompass prevalent diabetes cases. Additionally, this study is the first of its kind in Central Asia to anticipate the one-year mortality of diabetes patients, and thus contributes significant information to the existing body of literature on this topic. The analysis took into account comorbidities as well as demographic factors. These findings can help in the development of improved protocols and strategies to manage diabetes in healthcare settings, while also considering socio-demographic factors and cultural variations. Moreover, the results may aid in increasing community awareness campaigns and promoting healthy lifestyles to prevent diabetes mortality. Lastly, these results may be useful in initiating further research on the cost-effectiveness of diabetes management in order to assess the economic burden of the disease.

## Conclusion

This study developed a comprehensive machine learning platform to predict one-year mortality in patients with DM based on administrative health data. The results of the study showed that the constructed data-driven models can predict one-year mortality in DM patients with an AUC of more than 0.78, which is considered ‘fair’ (close to ‘good’) as per objective metrics of diagnostic tests. The study identified age, duration of diabetes, hypertension and sex as the top most important features. These findings could be used to develop better treatment protocols for diabetes patients that take into account socio-demographic and cultural factors. Additionally, the results would help increase community awareness campaigns and promote healthy lifestyles to prevent diabetes mortality.

Overall, this study demonstrates the potential for using machine learning to build accurate predictive models of one-year mortality in DM patients based solely on administrative health data. This focus is warranted because it can help healthcare practitioners to develop individualized treatment plans and interventions to mitigate adverse consequences for high-risk patients. Furthermore, it could aid in resource allocation, as high-risk patients may require more frequent monitoring or follow-up care. Integrating our findings with further information such as laboratory data, patients' medical history, and information on important comorbidities and anthropometric indices could potentially improve the performance of the predictive models in the future.

## Materials and methods

### Study population

In this dataset, patients with Type 1, Type 2, and other types of diabetes were included. The database was extracted from UNEHS based on International Classification of Diseases 10 (ICD-10) codes for diabetes (Type 1 DM: E10; Type 2 DM: E11). The UNEHS collects individual inpatient and outpatient electronic registries with clinical data. All of these patients were registered between 2014 and 2019. The study involved secondary data that was derived from the UNEHS. Therefore, the requirement for informed consent from study participants was waived by the Nazarbayev University Institutional Review Ethics Committee (NU-IREC 490/18112021). All methods were carried out in accordance with the “Reporting of studies conducted using observational routinely-collected health data” (RECORD) guideline. After cleaning and preprocessing the initial dataset, the final cohort consisted of 472,950 DM patients.

### Comorbidity selection

There are several key comorbidities that can affect diabetes mortality. Diabetes can lead to the development of cardiovascular diseases^52,53^, cerebrovascular accident (CVA), also known as stroke^54,55^ and chronic kidney disease^56^. In addition, diabetes is associated with obesity^57^ and hypertension^42,43^ with modifiable and non-modifiable risk factors. The UNEHS databases for hypertension^58^, CVA^59^, coronary heart disease and neoplasms were merged using patients’ unique population registry numbers to define comorbid conditions. Diabetes Mellitus (DM) and neoplasms, or tumors, have a complex relationship. While there is evidence to suggest that individuals with DM are at an increased risk for certain types of neoplasms, the underlying mechanisms are not yet fully understood. According to Zhu and Qu^60^, the risk of cancers appears to be increased in both type 1 diabetes mellitus (T1DM) and type 2 diabetes mellitus (T2DM). Cancer was also reported to be the second most common cause of death for people with T1DM.

### Model training, selection, and evaluation

We used nine classifiers: (1) Gaussian Naïve Bayes (GNB); (2) K-nearest neighbors (KNN); (3) logistic regression with *L*_*2*_ ridge penalty (LRR); (4) random forest (RF); 95) AdaBoost with decision trees (ADB); (6) gradient boosting with regression trees (GBRT); (7) XGBoost (XGB); (8) linear discriminant analysis (LDA); and (9) perceptron (PER). Table 5 shows the candidate values of hyperparameters that were used in the model selection phase for these classifiers.

**Table 5:** Hyperparameters space for grid search with cross-validation model selection.

Classifiers	Hyperparameter	Candidate hyperparameter space
Gaussian Naive Bayes (GNB)	–	–
K Neighbors Classifier (KNN)	Number of neighbours	3, 5
Logistic Regression (LRR)	Penalty	*L*_*2*_
	Regularization parameter C	100, 10, 1.0, 0.1, 0.01
Random Forest Classifier (RFC)	Number of estimators	10, 100, 1000
	Maximum depth	2, 5, 10, 20, 50
	Maximum features	'auto', 'sqrt', 'log2'
Ada Boost Classifier (ADB)	Number of estimators	10, 100, 1000
	Learning rate	0.001, 0.01, 0.1
Gradient Boost Classifier (GBC)	Number of estimators	10, 100, 1000
	Learning rate	0.001, 0.01, 0.1
XGBoost Classifier (XGB)	Maximum depth	5, 10, 100
	Number of estimators	10, 100, 1000
	Learning rate	0.001, 0.01, 0.1
Linear Discriminant Analysis (LDA)	Solver	‘svd’, ‘lsqr’, ‘eigen’
	Tolerance	0.00001, 0.0001, 0.0003
Perceptron (PER)	Alpha	0.0001, 0.001, 0.01
	Penalty	*L*_*2*_,* L*_*1*_, None

In this study, the choice of prediction models was based on several principles. First, we selected model types that cover five commonly known groups: ensemble, Gaussian process, nearest neighbor, linear models, and discriminant analysis. Second, these models have been used extensively in previous studies to predict comorbidities of diabetes, preliminary diagnosis of diabetes, and mortality rate.

Many of our models were used previously for predicting ICU admissions of COVID-19 patients^61^. LDA has been deployed for predicting diabetes through fatty biomarkers in blood^62^. KNN was used to predict diabetes risk of de-identified patients from the Vanderbilt University Medical Center (VUMC) through the use of the Medical Information Mart for Intensive Care III (MIMIC-III) dataset^63^. GBM, XGBoost, AdaBoost, LR, and RF were utilized to predict one-year mortality rate in heart transplantation patients, including those with diabetes mellitus^64^^.^ Similarly, other researchers used random forest and logistic regression to predict mortality rate in diabetic ICU patients^13^.

Studies based on the Istituto Clinico Scientifico Maugeri in Italy predicted diabetes complications using LR, NB, and RF^65^. A similar problem was addressed by other researchers that showed the superiority of XGBoost^12^. The XGBoost itself is considered as one of the best predictive models for tabular data, and it has been widely used in Kaggle competitions^66^. In our case, XGBoost and GBRT showed the best performance.

**Figure 3:**
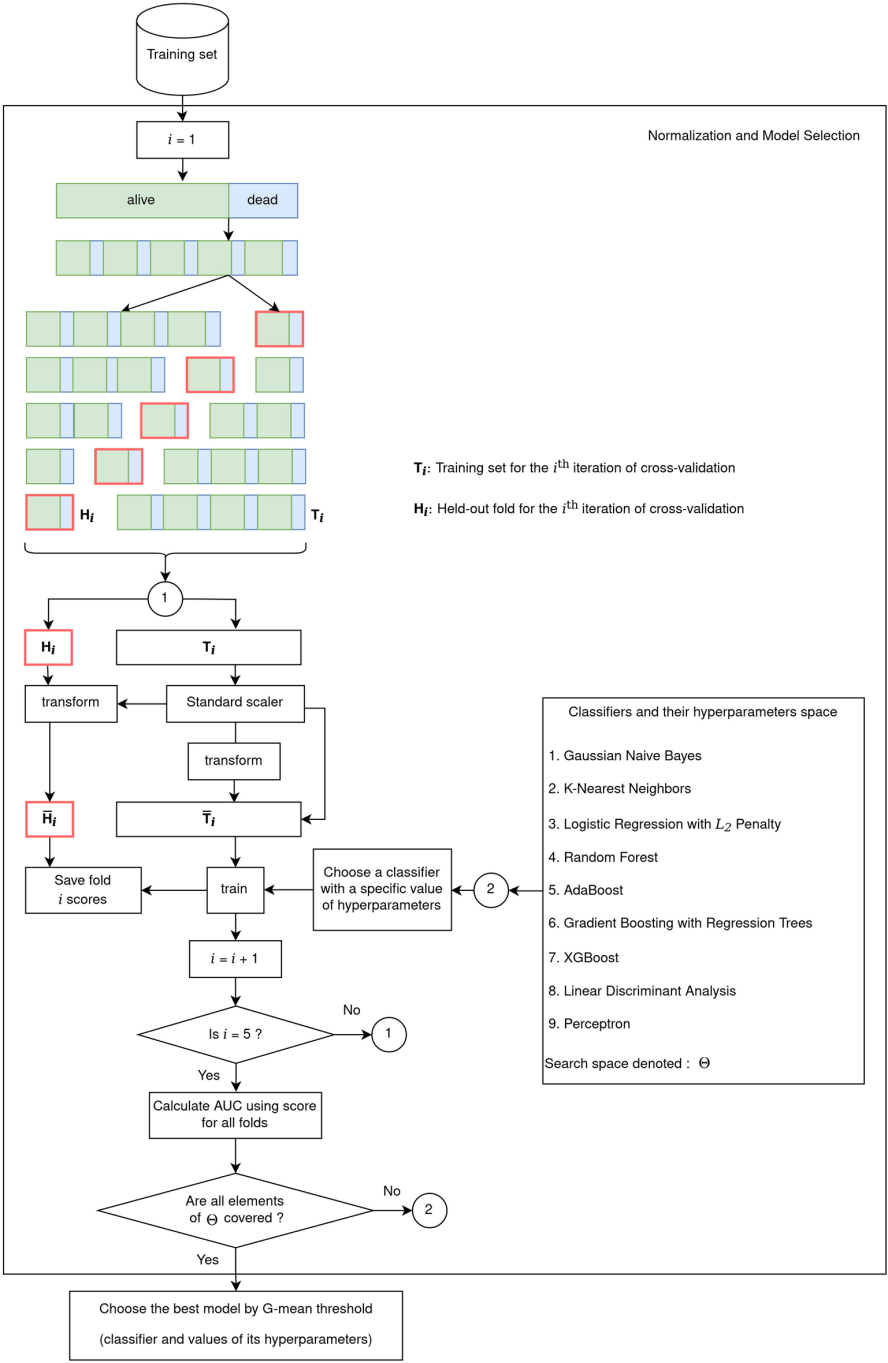
A schematic diagram of the implemented model selection with 5-fold cross-validation.

The yearly-specific model selection was performed using stratified 5-fold cross-validation (5-CV) applied to each yearly-specific training set. Figure 3 shows a schematic diagram of the model selection procedure using a 5-fold CV. The stratification is performed to keep the proportion of samples that appear in each fold the same as the original data. This practice gives a better view of the classifier performance in situations when the prior probability of classes is the same as their proportions within the data at hand. Furthermore, in each iteration of 5-CV, we standardize each feature based on the training data used in that iteration (i.e., full training data with one fold excluded). In this regard, we subtract the mean of that feature and divide it by its standard deviation. This way the feature vector is centered around zero and will have a standard deviation of one. The statistics obtained from the iteration-specific training data are then used to normalize the held-out data in the excluded fold. The selection of a year-specific classifier is based on the AUC metric, which is independent of any specific decision threshold used in the classifier^67^. As a result, the decision threshold of selected classifiers is further tuned using training data to maximize the geometric mean of sensitivity and specificity (G-mean). This is in contrast with the usual practice of relying on a classifier “default” decision threshold, which may lead to low G-mean values for highly imbalanced datasets such as ours.

The best year-specific classification rule and the values of its hyperparameters that were identified from the 5-CV model selection were used to train one final year-specific classifier on the entire training set after normalization. To normalize the entire training set, the same normalization that was used in each iteration of 5-CV was used. For prediction and evaluation on the test set, the statistics that were obtained on the training set are used to normalize each observation in the test set before using it as the input for the classifier.

### Software and packages

The computations were performed using a virtual server with an AMD Opteron Processor 6174-2.19 GHz with 22 processors, 200 GB of RAM, and storage 3.9 TB, running Windows Server 2019 Standard (64 bit) operating system. The main program was implemented in Python (version 3.10; Python Software Foundation) using open-source packages including sklearn (version 1.1.2), xgboost, numpy, pandas, seaborn, matplotlib, and shap.

### Ethics approval and consent to participate

The study was approved by the Nazarbayev University Institutional Review Ethics Committee (NU-IREC 203/29112019), with exemption from informed consent.

## Supplementary Information

Supplementary Information 1.

Supplementary Information 2.

## Data Availability

The data that support the findings of this study are available from the Republican Center for Electronic Health of the Ministry of Health of the Republic of Kazakhstan but restrictions apply to the availability of these data, which were used under license for the current study, and so are not publicly available. Data are however available from the corresponding author, Gaipov A., upon reasonable request and with permission of the Ministry of Health of the Republic of Kazakhstan.
