# Supplementary material for: Predicting 1-year mortality of patients with diabetes mellitus in Kazakhstan based on administrative health data using machine learning
Source: Sci Rep. 2023 May 24;13:8412. doi: 10.1038/s41598-023-35551-4 (PMC10206549; doi:10.1038/s41598-023-35551-4)
Supplement: Supplementary file 1 — Supplementary Information 1. [file 41598_2023_35551_MOESM1_ESM.pdf]

Predicting One-year Mortality of Patients with Diabetes Mellitus in Kazakhstan based on Administrative Health Data using Machine Learning

Aidar Alimbayev[1,2], **Gulnur Zhakhina**[2], **Arnur Gusmanov**[2], **Yesbolat Sakko**[2], **Sauran Yerdessov**[2], **Iliyar Arupzhanov**[1], **Ardak Kashkynbayev**[3], **Amin Zollanvari**[1], **and Abduzhappar Gaipov**[2,*]

[1]School of Engineering and Digital Sciences, Nazarbayev University, Kabanbay Batyr Avenue 53, Astana, Kazakhstan
[2]Department of Medicine, Nazarbayev University School of Medicine, Kerey and Zhanibek Khans Street 5/1, Astana, Kazakhstan
[3]Department of Mathematics, Nazarbayev University, Kabanbay Batyr Avenue 53, Astana, Kazakhstan
*abduzhappar.gaipov@nu.edu.kz

Supplementary Materials

Supplementary figures.

SHAP Analysis plots: In the following figures, a red dot in plots on the left shows a high value of the feature for a patient, whereas a blue dot shows a low value. The likelihood of mortality increases (decreases) for a positive (negative) SHAP value. Positive SHAP values for red dots show a direct dependence of the feature and the outcome, whereas the same values for blue dots imply an inverse dependence. The plots on the right show the feature importance (the mean absolute SHAP value) on outcome prediction made by the model (a longer bar shows a more important feature).

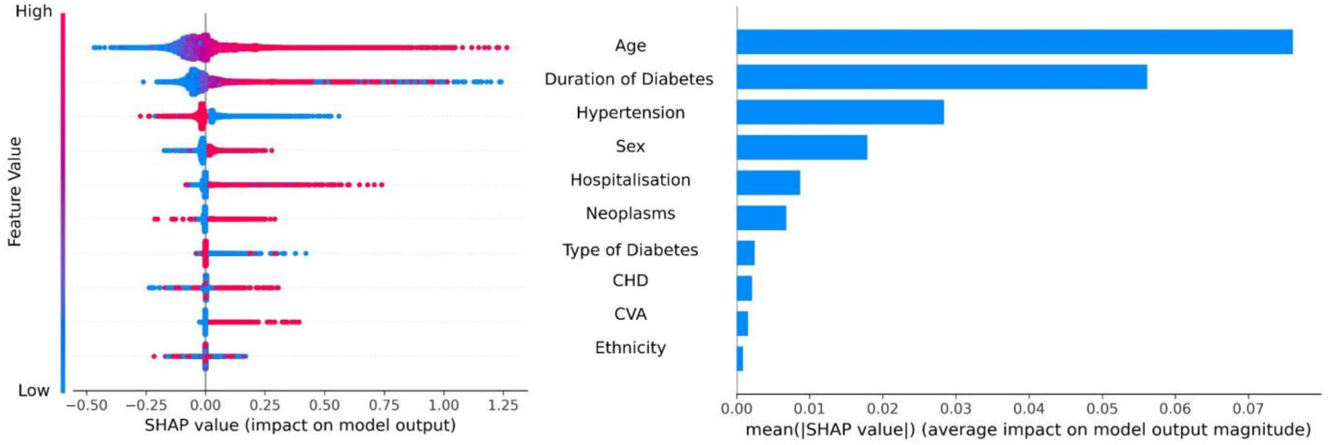

(a) (b)

Supplementary figure S1. SHAP analysis of 2017-specific cohort: (**a**) SHAP summary dot plot and (**b**) the mean absolute SHAP value bar plots for 2017-specific cohort.

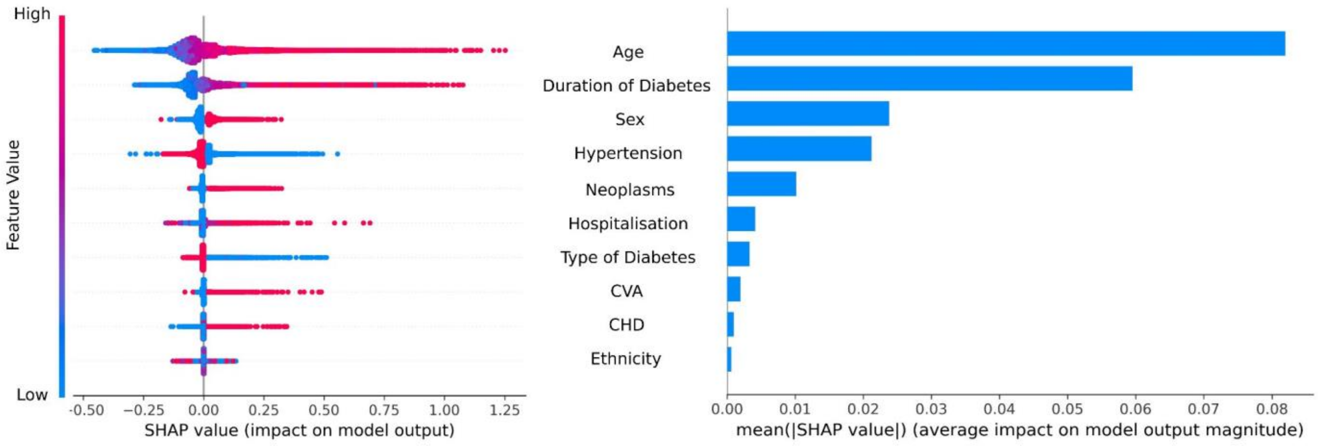

(a) (b)

Supplementary figure S2. SHAP analysis of 2018-specific cohort: (**a**) SHAP summary dot plot and (**b**) the mean absolute SHAP value bar plots for 2018-specific cohort.

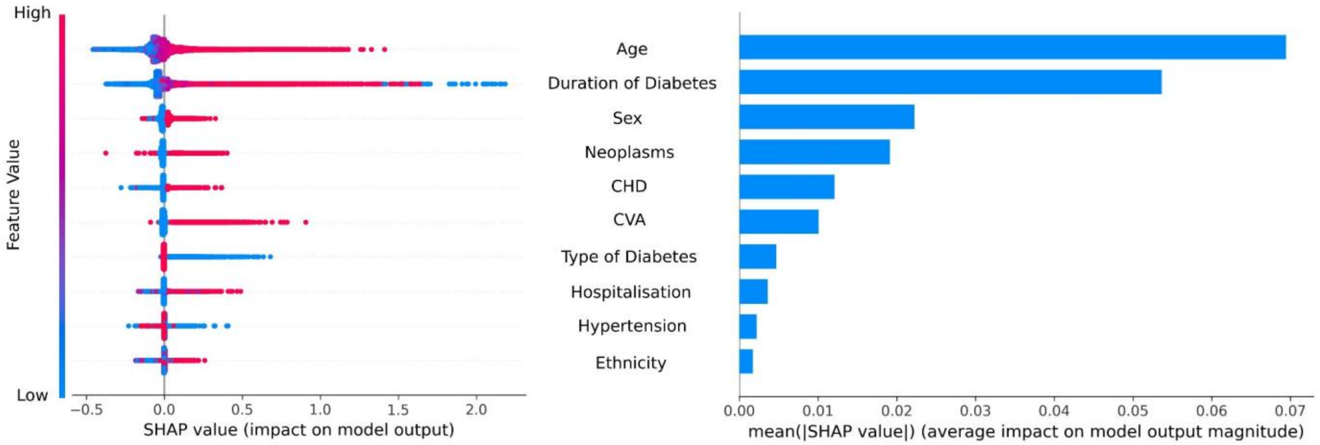

(a) (b)

Supplementary figure S3. SHAP analysis of 2019-specific cohort: (**a**) SHAP summary dot plot and (**b**) the mean absolute SHAP value bar plots for 2019-specific cohort.

Description of the subcohort selection: Fig. S4 depicts six types of patient groups in our dataset. A triangle indicates the date of diabetes diagnosis, while a circle indicates the exit date, which signifies the death of a patient. For instance, we choose the year 2018 as the year of observation. The subcohort for 2018 is made out of two patient groups. The first group (case group) includes patients who were diagnosed before the beginning of 2018 and died during that year (similar to case 1 in the figure, which is identified by a grey line and markers). The second group (control group) comprises patients diagnosed before the beginning of 2018 but were still alive during that year (similar to case 2 and 4, which are also highlighted by grey lines and markers). Patients who died before the start of 2018 were excluded from the subcohort (case 3 in the figure). Similarly, those who were diagnosed with diabetes mellitus during 2018 were excluded from the subcohort either (case 5 and 6 in the figure). Only patients with available clinical information and who were alive up to the end of 2017 were included. Therefore, we chose subcohorts for 2018 and predicted one-year mortality for 337,846 patients out of a total of 472,950 patients. It is noteworthy that diabetes can occur much earlier than the diagnosis date.

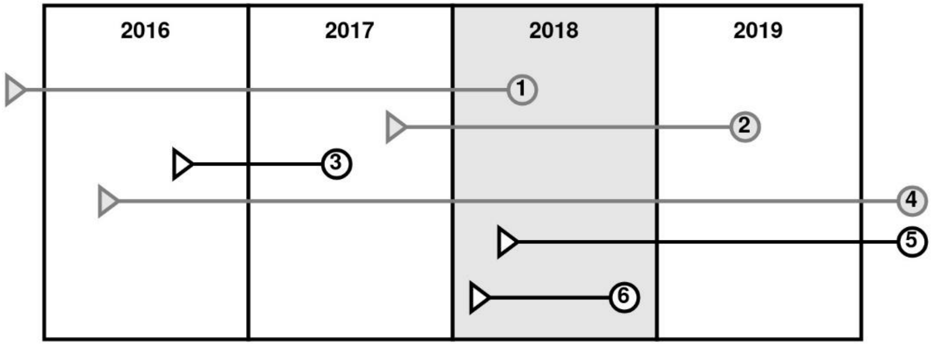

Supplementary figure S4. Description of subcohort selection

Feature description:

Feature	Description	Unit	Type	Values/Range	Mean
Age	age at the first hospitalisation recorded in the database	years	numeric	min - 9 days<br>max - 99.33 years	56.48 years
Sex	gender: female or male	binary	categorical	0 - female<br>1 - male	0.36
Ethnicity	three major categories: Kazakhs, Russians and others	tertiary	categorical	1 - Kazakh<br>2 - Russian<br>3 - other	1.74
Type of Diabetes	type of diabetes: T1D or T2D, and other types	tertiary	categorical	1 - type 1 diabetes<br>2 - type 2 diabetes<br>3 - other types of diabetes	1.93
CHD	comorbidity for diabetes (yes/ no)	binary	categorical	0 - no<br>1 - yes	0.27
CVA	comorbidity for diabetes (yes/ no)	binary	categorical	0 - no<br>1 - yes	0.030
Neoplasms	comorbidity for diabetes (yes/ no)	binary	categorical	0 - no<br>1 - yes	0.019
Hypertension	comorbidity for diabetes (yes/ no)	binary	categorical	0 - no<br>1 - yes	0.85
Hospitalisation	number of hospitalizations during observation period	frequency	numeric	min - 0<br>max - 32	0.41
Duration of Diabetes	follow-up time until December 31 the preceding year of prediction	years	numeric	min - 1 day<br>max - 47.03 years	5.59 years

CHD - coronary heart disease; CVA - cerebrovascular accident; T1D - type 1 diabetes; T2D - type 2 diabetes

Supplementary Table S1. Features description

	Predicted		
Actual		Negative	Positive
	Negative	TN: 32794	FP: 30191
	Positive	FN: 318	TP: 2250

TN – True Negative; FP – False Positive; FN – False Negative; TP – True Positive

Supplementary Table S2. Confusion matrix for 2016-year cohort test set

	Predicted		
Actual		Negative	Positive
	Negative	TN: 36416	FP: 36084
	Positive	FN: 349	TP: 2542

TN – True Negative; FP – False Positive; FN – False Negative; TP – True Positive

Supplementary Table S3. Confusion matrix for 2017-year cohort test set

	Predicted		
Actual		Negative	Positive
	Negative	TN: 36474	FP: 44694
	Positive	FN: 330	TP: 2963

TN – True Negative; FP – False Positive; FN – False Negative; TP – True Positive

Supplementary Table S4. Confusion matrix for 2018-year cohort test set

	Predicted		
Actual		Negative	Positive
	Negative	TN: 31502	FP: 57820
	Positive	FN: 213	TP: 3167

TN – True Negative; FP – False Positive; FN – False Negative; TP – True Positive

Supplementary Table S5. Confusion matrix for 2019-year cohort test set

Figure and table legends

Supplementary figure S1. SHAP analysis of 2017-specific cohort: (**a**) SHAP summary dot plot and (**b**) the mean absolute SHAP value bar plots for 2017-specific cohort.

Supplementary figure S2. SHAP analysis of 2018-specific cohort: (**a**) SHAP summary dot plot and (**b**) the mean absolute SHAP value bar plots for 2018-specific cohort.

Supplementary figure S3. SHAP analysis of 2019-specific cohort: (**a**) SHAP summary dot plot and (**b**) the mean absolute SHAP value bar plots for 2019-specific cohort.

Supplementary figure S4. Description of subcohort selection

Supplementary Table S1. Features description

Supplementary Table S2. Confusion matrix for 2016-year cohort test set

Supplementary Table S3. Confusion matrix for 2017-year cohort test set

Supplementary Table S4. Confusion matrix for 2018-year cohort test set

Supplementary Table S5. Confusion matrix for 2019-year cohort test set
