# Supplementary material for: Predicting 1-year mortality of patients with diabetes mellitus in Kazakhstan based on administrative health data using machine learning
Source: Sci Rep. 2023 May 24;13:8412. doi: 10.1038/s41598-023-35551-4 (PMC10206549; doi:10.1038/s41598-023-35551-4)
Supplement: Supplementary file 2 — Supplementary Information 2. [file 41598_2023_35551_MOESM2_ESM.docx]

Supplementary material provides the results of SHAP analysis of 2017-, 2018-, and 2019- cohorts, as well as the rationale behind the selection of subcohorts.
